# Application and exploration of virtual laboratory platform in standardized residency training for clinical laboratory physicians

**DOI:** 10.1186/s12909-026-10159-0

**Published:** 2026-08-18

**Authors:** Dan Zhao, Lin Lin, Shijun Li, Dapeng Ding

**Affiliations:** Department of Clinical Laboratory Medicine, The First Affiliated Hospital of Dalian Medical University, Dalian, Liaoning P.R. China

**Keywords:** Clinical laboratory, Residents, Standardized residency training, Virtual laboratory, Virtual simulation technology

## Abstract

**Background:**

Standardized residency training for clinical laboratory residents is essential for cultivating high-quality laboratory professionals. Virtual simulation technology has demonstrated significant advantages in education, yet its application in standardized residency training remains insufficiently explored. This study aimed to develop and evaluate a virtual laboratory platform to enhance residents’ clinical skills and practical competencies.

**Methods:**

A virtual laboratory platform was constructed using the Unity3D engine, comprising a virtual experiment operations module and a regular training content module, grounded in experiential learning, deliberate practice, and constructivism theories. A randomized controlled trial was conducted involving 68 residents with no prior PCR experience. Participants were randomly assigned to an experimental group (using the virtual platform for TB PCR detection training) or a control group (receiving traditional instruction). Outcomes were assessed via a 100-point composite score (50% theory, 50% practical skills) and a validated questionnaire (CVI = 0.92, Cronbach’s α = 0.842).

**Results:**

The virtual laboratory platform, covering five core subspecialties, was successfully developed. The experimental group significantly outperformed the control group in theoretical scores (47.03 ± 2.59 vs. 44.98 ± 3.15, *P* = 0.006), skill scores (46.74 ± 2.78 vs. 45.17 ± 3.02, *P* = 0.031), and total scores (93.77 ± 4.06 vs. 90.15 ± 5.44, *P* = 0.003). Questionnaire results showed high satisfaction: 97.1% agreed that the content was systematic, 92.6% reported increased interest and learning efficiency, and 91.2% affirmed comprehensiveness and autonomous learning support, with an overall satisfaction rate of 91.2%.

**Conclusions:**

The virtual laboratory platform improved residents’ theoretical knowledge and practical skills, and received highly positive feedback. Our findings suggest it represents a promising tool for enhancing the quality of standardized residency training in clinical laboratory medicine.

**Supplementary Information:**

The online version contains supplementary material available at https://doi.org/10.1186/s12909-026-10159-0.

## Introduction

Standardized residency training (hereinafter referred to as “residency training”) is crucial for cultivating high-level professionals [1, 2]. Medical laboratory science is a comprehensive, technical, and highly practical discipline that serves as a bridge between basic medicine and clinical medicine. The quality of residency training for laboratory residents directly impacts the direction and future development of laboratory medicine, as well as the quality of medical services [3, 4]. However, despite these efforts, laboratory residency training—a relatively young specialty—faces persistent challenges. First, while experienced laboratory experts are abundant, there is a notable imbalance in teaching resources. Some instructors lack the necessary teaching experience, clinical reasoning skills, and effective communication strategies, which affects the overall effectiveness of training [5]. Furthermore, grassroots and small-to-medium residency training bases are poorly equipped, limiting trainees’ hands-on practice with costly advanced technologies such as second-generation sequencing and mass spectrometry and hindering their proficiency in these sophisticated tools [6, 7]. Additionally, the absence of a robust management system for training facilities, combined with disorganized content and unclear learning paths and evaluation standards, prevents laboratory residents from achieving a deep understanding of the training content [8].

Simulation-based education has become a powerful adjunct to clinical training, as it provides standardized, repeatable, and risk-free learning environments conducive to deliberate practice [9]. Among these modalities, virtual reality (VR) and simulation are increasingly recognized as valuable tools to address key challenges in medical education, offering particular promise for enhancing practical skills training in specialized fields like laboratory medicine. Virtual laboratory platforms can provide standardized, accessible, and safe training environments that are difficult to replicate with traditional methods [10]. The immersive and interactive nature of VR can improve learner engagement and motivation, while allowing for repetitive practice and personalized feedback crucial for mastering complex laboratory procedures [11]. Furthermore, recent developments in collaborative virtual reality (CVR) suggest opportunities to enhance teamwork and communication skills within virtual laboratory settings, mirroring real-world clinical laboratory workflows [12]. As residency programs seek to optimize training and equip future laboratory physicians with essential competencies, the rigorous development and evaluation of innovative virtual laboratory platforms are of paramount importance.

In recent years, immersive virtual simulation has been preliminarily applied in laboratory medicine education, covering microbial detection, molecular diagnostic testing, biosafety training, clinical biochemistry practice, and blood-related laboratory procedural training scenarios [13–17]. Existing studies have verified that virtual training can effectively reduce experimental operation risks, compensate for shortages of clinical specimens and expensive consumables, and improve students’ learning initiative and long-term skill retention. However, most currently reported laboratory virtual teaching tools focus only on independent or single experimental modules, lacking systematic, multi-discipline virtual training systems oriented to the entire standardized residency training process. At present, there is still a lack of comprehensive immersive virtual platforms covering all core subspecialties of clinical laboratory medicine, and empirical evaluations focusing specifically on laboratory resident training remain insufficient.

Against this background, our team constructed a comprehensive five-subspecialty immersive virtual laboratory platform oriented to standardized residency training. Different from previous single-module virtual teaching tools, this platform integrates systematic training resources, standardized operation processes, and multi-scene simulation environments, aiming to resolve the long-standing dilemmas of insufficient practical opportunities, high training costs, unstandardized teaching procedures, and uneven teaching quality in traditional residency training. On this basis, the present study aimed to evaluate the application effect of this virtual platform in residency teaching, providing an evidence-based optimization strategy for improving the quality of clinical laboratory standardized training.

## Methods

### Design of the virtual laboratory intervention

#### Development of the virtual laboratory platform and interaction design

To provide a high-fidelity training environment, the platform was developed using the Unity3D engine, with system logic implemented via C# programming. Professional 3D modeling software, including 3ds Max and Maya, was employed to construct immersive laboratory settings. The system architecture was designed to support real-time scene optimization and a variety of intuitive interaction mechanisms, such as clicking, dragging, and sliding. These design choices aim to facilitate an active learning experience by allowing residents to engage directly with the virtual space and perform complex experimental workflows. The overall framework was established to integrate two primary components: a virtual experiment operations module and a regular training content module.

#### Educational theoretical basis

This virtual laboratory platform is constructed based on multiple classic educational theories to ensure the scientific rigor and standardization of the training design. The virtual experiment operations module is developed following Experiential Learning Theory [18] and Deliberate Practice Theory [19]. By adopting immersive simulated experimental scenarios, hierarchical training and assessment modes, and stepwise operational guidance, this module enables residents to enhance professional proficiency through hands-on practical experience. The regular training content module integrates Constructivism [20]. Equipped with diversified hierarchical learning resources and a closed-loop evaluation system, this module supports residents’ personalized and active knowledge construction, thereby consolidating their theoretical foundation and progressively addressing individual competency gaps. Together, the two modules synergistically empower residency training, ensuring a scientifically sound and standardized training system.

#### Functional design and scope of the virtual experiment operations module

The virtual experiment operations module was designed to systematically simulate the professional environments of five core disciplines in clinical laboratory medicine: Clinical Hematology and Body Fluid, Clinical Biochemistry, Clinical Immunology, Clinical Microbiology, and Clinical Molecular Diagnostics. The module’s scope was defined to encompass a full spectrum of laboratory procedures, ranging from routine specimen testing to advanced technologies such as mass spectrometry and second-generation sequencing. To support the pedagogical goal of progressive skill acquisition, the module incorporates a dual-mode instructional strategy: practice mode and assessment mode. The practice mode was designed to provide step-by-step guidance and immediate explanations to reinforce procedural understanding. In contrast, the assessment mode focuses on independent execution, featuring automated scoring and error analysis to help residents identify and rectify technical deficiencies. Interactive learning questions and digitized operation manuals were embedded within the modules to ensure that key learning objectives are addressed throughout the simulation.

#### Regular training content module

This module provides a diverse repository of learning resources, comprising four key components: (1) Morphological identification and image analysis of blood cells, microbes, and antinuclear antibodies; (2) Interpretation of common and specialized laboratory reports essential for clinical practice; (3) Case studies to enhance residents’ problem-solving skills; and (4) Educational materials and video resources that cover foundational theories, recent developments, and practical applications in laboratory medicine. Additionally, the module includes an assessment component for residency training, presented in the form of a question bank.

### Study participants and randomization

A total of 68 residents undergoing standardized training in the Department of Clinical Laboratory at the First Affiliated Hospital of Dalian Medical University were enrolled in this study. Participant recruitment was conducted between September 1 and September 15, 2024. The inclusion criteria were: (1) active residents currently registered in the national standardized residency training program within our specialty, and (2) voluntary participation with informed consent. The exclusion criteria were any prior practical training, hands-on performance, or clinical certification in diagnostic PCR experimental procedures, thereby ensuring that all participants shared an identical baseline of zero prior practical experience with PCR techniques. Notably, the study cohort included all eligible residents across different training grades during the 2024–2025 academic year, representing a full-population census rather than selective sampling.

Participants were randomized in a 1:1 ratio via a computer-based random sequence generator. Group allocation assignments were maintained in sealed, opaque envelopes handled by an independent departmental secretary who had no involvement in resident instruction or grading. Due to the obvious visual differences between operating a laptop-based virtual platform and attending a physical laboratory demonstration, complete double-blinding of the residents and instructors was logistically impossible. However, to minimize evaluation bias, grading was performed based on a standardized scoring rubric by senior instructors who were entirely blinded to the residents’ group assignments.

### Instructional procedure

The main challenge in integrating a virtual laboratory platform into residency training lies in demonstrating its efficacy in improving the quality of training. To address this, we designed a pedagogical experiment focused on polymerase chain reaction (PCR) detection of Mycobacterium tuberculosis (TB). Experimental Group: Each participant in the experimental group accessed the TB PCR detection module by logging into the virtual laboratory platform using a laptop. They then performed the following steps: (1) Loaded the course and selected “Practice Mode”. (2) Viewed the “TB PCR Detection Experiment Operation Manual” to familiarize themselves with the experimental objectives, principles, procedural steps, and necessary reagents and instruments. (3) Engaged in simulated practical exercises, which included sample processing, nucleic acid extraction, amplification, and result interpretation. Participants were permitted to access the system and repeat exercises as needed. Control Group: Participants in the control group received traditional instruction, which consisted of explanation, demonstration, and hands-on practice. Specifically: (1) A 45-minute traditional PowerPoint-based classroom lecture was delivered to provide a comprehensive overview of the experimental principles and methodologies of TB nucleic acid amplification experiments. (2) The instructor conducted a 30-minute physical demonstration of the TB PCR procedure. (3) Participants were organized into smaller groups to carry out the experiment with simulated samples.

### Evaluation and statistical analysis

#### Resident evaluation

Resident evaluation was divided into two parts. The first part was a post-course evaluation of teaching effectiveness. This included assessments of their theoretical knowledge of nucleic acid detection principles and methods, as well as their practical skills in sample handling, nucleic acid extraction, and result interpretation. The assessment was graded on a total score of 100 points, with theoretical knowledge and practical skills each accounting for 50% of the overall grade.

The second part was the collection of residents’ opinions through a questionnaire. The questionnaire was developed following a review of literature related to standardized residency training [1] and virtual simulation education [17]. The items were designed to assess key educational outcomes as suggested by previous studies on virtual learning environments [21].

The survey was scored on a standard 3-point Likert scale (ranging from “Disagree” to “Agree”, with “Neutral” as an intermediate option). To guarantee scientific validity, the instrument underwent formal content validation by a panel of 3 senior laboratory medicine educational experts (Content Validity Index, CVI = 0.92) and was pilot-tested on a small independent cohort of 10 students (excluded from the final analysis) prior to formal execution. The internal consistency reliability of the questionnaire was assessed using the formal study dataset, yielding a Cronbach’s alpha coefficient of 0.842, which confirms the strong reliability and psychometric soundness of the instrument. After the test, all 68 participants had access to the virtual laboratory platform and completed a post-study questionnaire.

#### Statistical methods

Statistical analyses were performed using R software (version 4.3.2). Prior to inferential analysis, continuous variables were assessed for normality using the Shapiro-Wilk test. Measurement data that exhibited a normal distribution were reported as mean ± standard deviation (mean ± SD), and inter-group comparisons were conducted using the independent samples t-test. For the qualitative component, questionnaire survey results were expressed as frequencies and percentages (n, %), with the chi-square test (or Fisher’s exact test, where appropriate) used for inter-group comparisons. To determine the substantive educational significance beyond simple statistical p-values, Cohen’s d effect sizes along with their corresponding 95% confidence intervals (95% CIs) were calculated for the primary outcomes using the “effsize” package. The magnitude of the effect size was interpreted based on standard benchmarks: small (0.20 ≤ d < 0.50), medium (0.50 ≤ d < 0.80), and large (d ≥ 0.80). All statistical tests were two-tailed, and a p-value of less than 0.05 was deemed statistically significant.

## Results

### Successful construction and functional implementation of the virtual laboratory platform

**Fig. 1:**
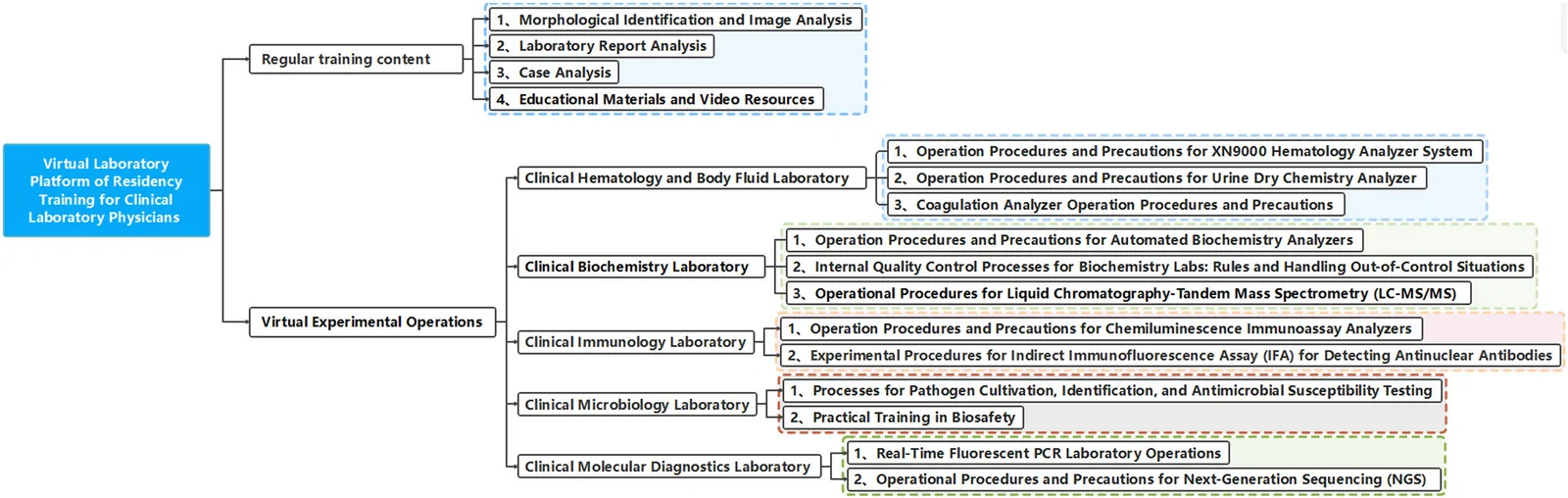
Content of the two modules of the virtual laboratory platform

The research successfully developed a comprehensive, immersive virtual laboratory platform tailored for standardized residency training. The platform’s architecture and the specific content of its two primary modules—the virtual experiment operations module and the regular training content module—are illustrated in Fig. 1.

### High-fidelity 3D environment and interactive features

**Fig. 2:**
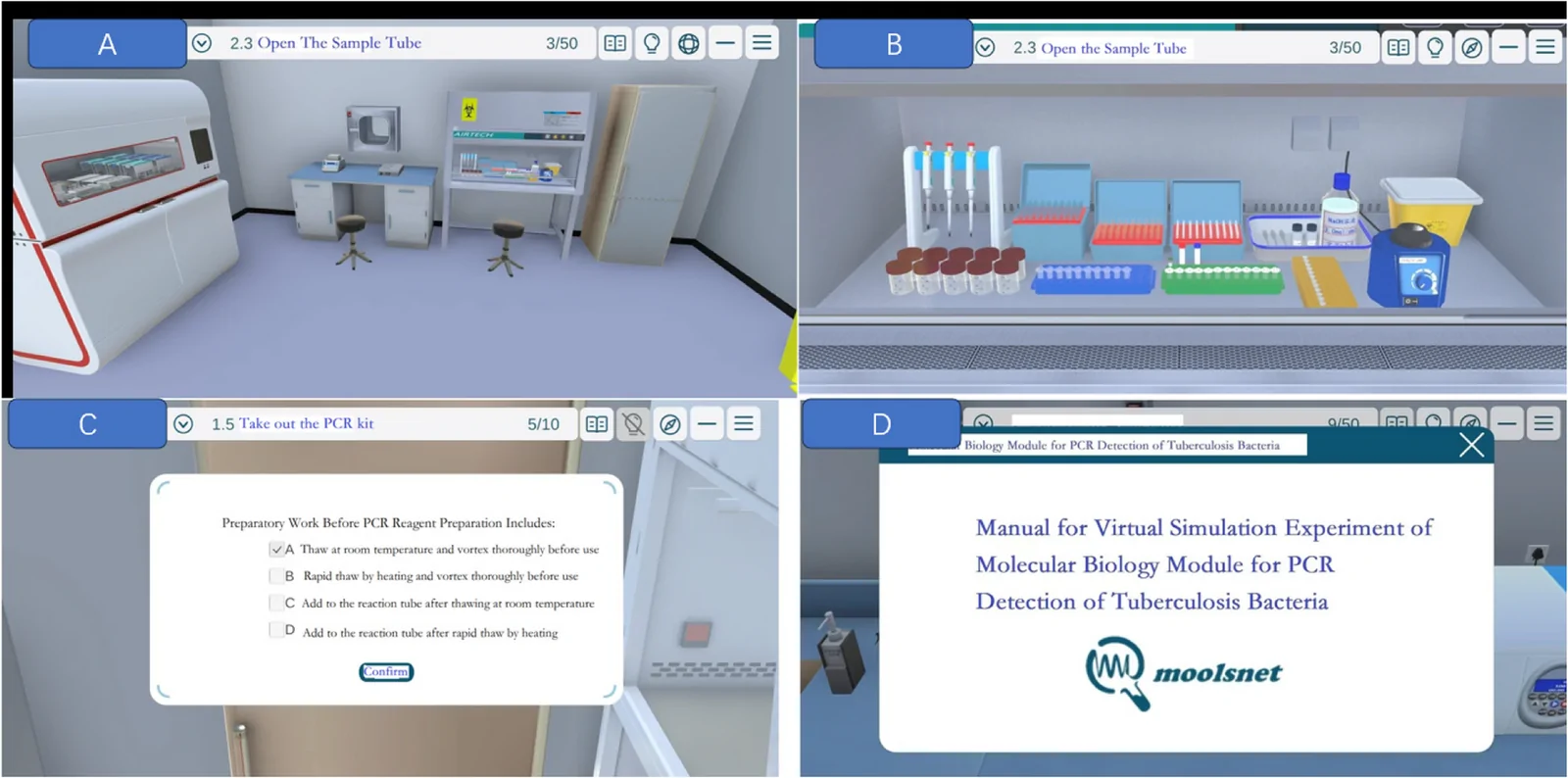
Virtual experimental operations module. **A** and **B **Realistic training milieu. **C **Learning questions. **D **Operation manual

As a primary outcome of the development process, the platform provides a high-fidelity, three-dimensional training milieu that closely replicates real-world clinical laboratory settings (Fig. 2A and B). The system successfully integrates intuitive interaction mechanisms, allowing residents to perform complex experimental steps through clicking and dragging. To ensure standardized learning, the platform features embedded pedagogical aids, including carefully curated learning questions targeting key objectives (Fig. 2C) and detailed, digitized operation manuals that guide users through each procedural stage (Fig. 2D).

### Discipline coverage and adaptive learning modes

**Fig. 3:**
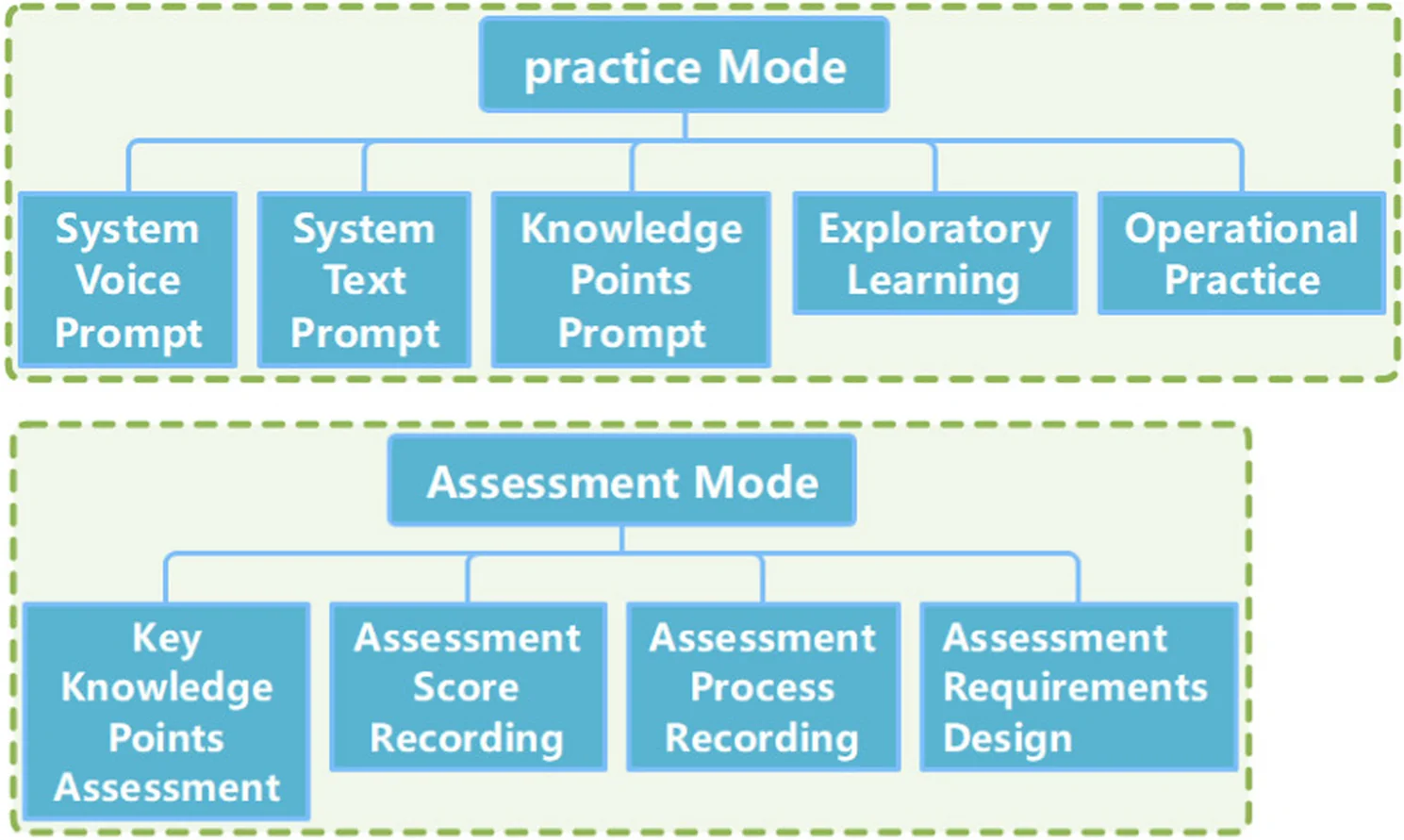
Practice and assessment modes

The developed platform effectively covers five core subspecialties of laboratory medicine, supporting a wide range of simulations from routine specimen testing to advanced molecular techniques. A key functional achievement is the implementation of a flexible dual-mode system (Fig. 3): Practice Mode: Provides residents with a guided environment featuring step-by-step instructions and theoretical explanations to facilitate knowledge acquisition. Assessment Mode: Enables independent execution of laboratory workflows. This mode successfully automates performance evaluation, providing immediate scoring and error analysis, which allows residents to identify specific technical gaps in a risk-free environment.

### Quantitative evaluation of teaching effectiveness

Compared with the control group, residents in the experimental group achieved higher scores in theoretical knowledge, practical skills, and total scores, with statistically significant differences (all *P* < 0.05). Specifically, the effect size (Cohen’s d) for the total score was 0.75 (95% CI [0.25, 1.25], *P* = 0.003), indicating a substantial educational impact. Detailed results are presented in Table 1.

**Table 1:** Comparison of scores between the experimental and control groups (mean ± SD)

Assessment Module	Experimental Group (*n* = 34)	Control Group (*n* = 34)	t-value	*P*-value	Cohen’s d	95% Confidence Interval (95% CI)
Theoretical Score (Max: 50)	47.03 ± 2.59	44.98 ± 3.15	2.873	0.006	0.71	[0.21, 1.20]
Skill Score (Max: 50)	46.74 ± 2.78	45.17 ± 3.02	2.204	0.031	0.54	[0.05, 1.03]
Total Score (Max: 100)	93.77 ± 4.06	90.15 ± 5.44	3.060	0.003	0.75	[0.25, 1.25]

### Questionnaire survey results

All 68 residents completed the questionnaire (100% response rate). The results indicated that the virtual laboratory platform was well received. Specifically, 97.1% of residents agreed that the training content was systematic and logical, helping them master knowledge; 92.6% agreed that the training stimulated their learning interest and improved learning efficiency; and 91.2% believed the training was comprehensive and fostered autonomous learning. The overall satisfaction rate was 91.2%. Detailed results are shown in Table 2.

**Table 2:** Questionnaire evaluation of the virtual laboratory platform [*n* = 68, n (%)]

Evaluation items	Agree	Neutral	Disagree
Promote the mastery of knowledge	66(97.1)	2(2.9)	0(0)
Stimulate interest in learning	63(92.6)	5(7.4)	0(0)
Systematic and logical training contents	66(97.1)	2(2.9)	0(0)
Facilitate the autonomous Learning	62(91.2)	6(8.8)	0(0)
Comprehensiveness training contents	62(91.2)	6(8.8)	0(0)
Improve learning efficiency	63(92.6)	5(7.4)	0(0)
Overall satisfaction	62(91.2)	6(8.8)	0(0)

## Discussion

Standardized residency training for clinical laboratory residents is the core stage for cultivating compound and high-quality laboratory medical talents in China [22]. The officially issued “Standardized Resident Standardized Training Base Identification Criteria” and “Standardized Resident Standardized Training Content and Standards” [23] formulated unified and standardized training objectives and assessment requirements for laboratory residency education, providing clear guidance for cultivating qualified laboratory residents. Nevertheless, numerous practical deficiencies still exist in the actual implementation of current residency training, including imbalanced teaching resources, insufficient advanced operational training, non-standardized training and evaluation systems, and overloaded rotation schedules with limited learning time [23, 24]. Therefore, innovative teaching interventions were urgently needed to compensate for the shortcomings of traditional residency training and improve the overall training quality of laboratory residents.

Virtual simulation technology constructs highly immersive and interactive three-dimensional clinical laboratory scenarios through computer technology, activating multi-sensory participation of vision, hearing, and touch, and has become an effective auxiliary teaching method for modern medical education [14, 25].

In this study, a controlled teaching experiment and questionnaire survey were conducted among 68 residents to systematically verify the teaching effect of the self-developed virtual laboratory platform. The experimental results confirmed that residents in the virtual training group achieved significantly better theoretical mastery and practical operational performance than those receiving traditional training. The post-study questionnaire further demonstrated that more than 90% of residents recognized the systematic nature, operability, and learning flexibility of the platform, with excellent comprehensive learning satisfaction. Our overall experimental outcomes are consistent with previous controlled trials on virtual laboratory teaching [13, 26, 27], which collectively confirm that virtual simulation platforms can effectively improve the learning performance and subjective satisfaction of laboratory trainees. Notably, further quantitative effect size analysis revealed a discrepant improvement magnitude between different learning outcomes. The relatively smaller effect size for practical skills (Cohen’s d = 0.54) compared with theoretical knowledge (Cohen’s d = 0.71) indicated that virtual simulation exerts a more prominent advantage in improving residents’ cognitive mastery and theoretical workflow comprehension. Nevertheless, face-to-face physical hands-on training remains irreplaceable for refining operational proficiency. Collectively, these findings demonstrate that integrating the virtual laboratory platform into standardized residency training can effectively enhance residents’ learning engagement and optimize short-term learning outcomes, while the platform should serve as an accelerant to traditional practical instruction rather than a complete replacement.

Compared with previously reported virtual simulation tools for laboratory medicine education, the present platform offers notable systematic and functional innovations. Most existing VR training systems only support single-module simulation, covering isolated items such as microbial identification, biochemical detection, gene testing, or blood transfusion procedures, and lack targeted curriculum design for standardized residency training [13, 15–17]. In contrast, our platform integrated scenarios of five core laboratory subspecialties, covering a wide range of experimental procedures from basic to advanced, providing residents with systematic and comprehensive learning content that matches the systematic training requirements of laboratory residency programs. Beyond disciplinary comprehensiveness, this platform provides flexible dual learning modes to accommodate individualized learning demands. The practice mode provides detailed, step-by-step guidance to enhance understanding, while the assessment mode facilitates targeted identification of weaknesses and subsequent skill refinement. Unlike most conventional VR platforms that focus merely on procedural simulation, this system integrates standardized operation training, report interpretation exercises, clinical case analysis, educational materials, and professional knowledge question banks, forming a complete teaching framework that simultaneously cultivates practical skills, theoretical knowledge, and clinical reasoning skills. Moreover, its online accessible design removes limitations of time, space, and experimental conditions, allowing residents to conduct flexible, repeated simulation practice to consolidate skills, which delivers advantages over traditional instruction and fragmented single-function virtual training tools.

Cost efficiency is an essential advantage of virtual simulation in medical training. Traditional laboratory teaching incurs continuous recurring costs related to clinical specimens, chemical consumables, and instrument maintenance, while high-risk and high-cost experiments are difficult to repeat in routine training. Although virtual platforms require initial hardware and developmental investment, they effectively reduce long-term operational expenses by eliminating repeated consumable consumption and equipment loss [28–30]. Beyond residency training, the platform can be flexibly extended to undergraduate education and in-service training. Such multi-scenario resource sharing further improves resource utilization and overall cost-effectiveness.

This study still had several limitations. First, this investigation was conducted at a single training center with a relatively limited sample size, and the effectiveness evaluation only adopted TB molecular detection as the representative experimental module, which inevitably restricts the generalizability and stability of the research findings. Second, the existing evaluation outcomes mainly rely on residents’ self-reported learning experience and satisfaction, which may bring potential subjective reporting bias. Third, this preliminary evaluation only focused on short-term learning performance immediately after training, while lacking long-term follow-up data of residents’ post-training clinical competency. Fourth, virtual human-computer interaction cannot fully simulate complex on-site clinical communication, unexpected specimen abnormalities, and real laboratory emergency scenarios; therefore, virtual simulation training can only serve as an effective auxiliary tool rather than a complete replacement for offline clinical practice and face-to-face faculty guidance.

In future studies, we will expand the sample size and carry out multi-center verification, add more experimental module evaluation indicators, and establish a long-term tracking mechanism for training effects. At the same time, we will further optimize the collaborative virtual interaction function of the platform, build a multi-dimensional quantitative evaluation system including learning duration, operation standard rate, and error statistics, and integrate the platform training results into the resident graduation assessment system, so as to further improve the standardized and intelligent level of laboratory residency training.

## Conclusion

Integrating a virtual laboratory platform into the residency training curriculum for laboratory residents represents a significant pedagogical innovation. It enriches both teaching methodologies and content while affording learning flexibility. Residents can manage their study schedules autonomously and engage in repetitive practice within a highly realistic virtual environment, thereby refining their technical skills. The platform’s rich repository of learning resources further cultivates diagnostic reasoning. However, it is essential to acknowledge that a virtual platform cannot entirely supplant traditional training modalities. Direct clinical interaction and mentorship from experienced physicians remain indispensable for developing competent clinicians. Therefore, while maximizing the advantages of virtual laboratory platforms, we must prioritize their seamless integration with established training methods to ensure the holistic development of residents into proficient clinical laboratory physicians equipped with a strong theoretical foundation and extensive practical experience.

## Supplementary Information

Supplementary Material 1.

## Data Availability

The datasets used and/or analysed during the current study are available from the corresponding author on reasonable request.
