# Supplementary material for: Application and exploration of virtual laboratory platform in standardized residency training for clinical laboratory physicians
Source: BMC Med Educ. 2026 Aug 18;26:1850. doi: 10.1186/s12909-026-10159-0 (PMC13760892; doi:10.1186/s12909-026-10159-0)
Supplement: Supplementary file 1 [file 12909_2026_10159_MOESM1_ESM.pdf]

Questionnaire of the Virtual Laboratory Platform for Clinical Laboratory Residents

Dear Clinical Laboratory Residents,

We are conducting a survey to understand your experience and satisfaction with the virtual laboratory platform. Your feedback is crucial for us to improve and enhance the platform further. Please indicate your level of agreement with each statement by selecting "Agree," " Neutral ", or "Disagree."

1. **Does the virtual laboratory platform help you master the knowledge and skills required for your residency?**

   ☐ Agree

   ☐ Neutral

   ☐ Disagree

2. **Does the platform stimulate your interest in learning skills and knowledge related to clinical laboratory science?**

   ☐ Agree

   ☐ Neutral

   ☐ Disagree

3. **Are the training contents on the virtual laboratory platform organized in a systematic and logical manner?**

   ☐ Agree

   ☐ Neutral

   ☐ Disagree

4. **Does the platform facilitate your autonomous Learning?**

   ☐ Agree

   ☐ Neutral

   ☐ Disagree

5. **Do you think the training contents provided by the virtual laboratory platform for resident physicians are comprehensive?**

   ☐ Agree

   ☐ Neutral

   ☐ Disagree

6. **Has the platform improved your learning efficiency and helped you manage your study time more effectively?**

   ☐ Agree

   ☐ Neutral

   ☐ Disagree

7. **Overall, are you satisfied with the virtual laboratory platform as a training platform for resident physicians?**

   ☐ Agree

   ☐ Neutral

   ☐ Disagree

If you have any further suggestions for improving the virtual laboratory platform, please write them below.

Thank you for taking the time to complete this survey. Your feedback will be invaluable in helping us improve the virtual laboratory platform to better meet your needs and optimize your learning experience.
